# Insights into the Mechanism of Supramolecular Self-Assembly in the *Astragalus membranaceus*–*Angelica sinensis* Codecoction

**DOI:** 10.1021/acsami.3c09494

**Published:** 2023-10-04

**Authors:** Pan Liang, Tao Bi, Yanan Zhou, Yining Ma, Xinyue Liu, Wei Ren, Sijin Yang, Pei Luo

**Affiliations:** † State Key Laboratories for Quality Research in Chinese Medicines, Macau University of Science and Technology, Macau 999078, China; ‡ National Traditional Chinese Medicine Clinical Research Base and Drug Research Center of Integrated Traditional Chinese and Western Medicine, The Affiliated Traditional Chinese Medicine Hospital of Southwest Medical University, Luzhou 646000, China

**Keywords:** supermolecules, codecoction, Astragalus membranaceus–Angelica sinensis, self-assembly, myocardial fibrosis, endothelial-to-mesenchymal transition

## Abstract

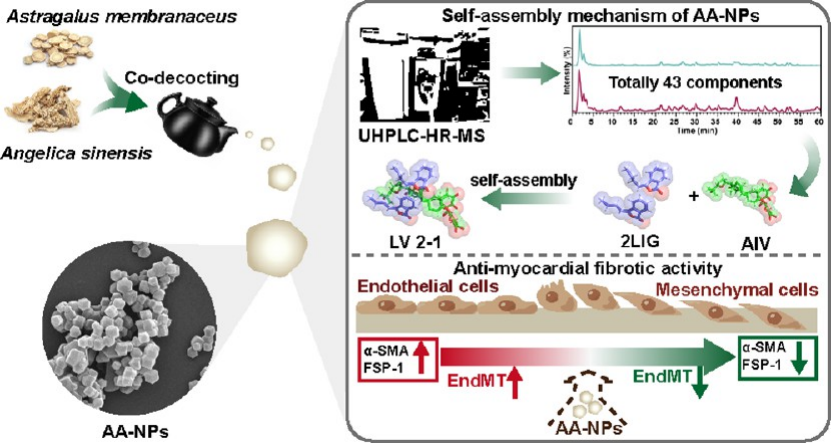

*Astragalus membranaceus* (Fisch.) Bge. (AM) and *Angelica sinensis* (Oliv.) Diels (AS) constitute a classic herb pair in prescriptions to treat myocardial fibrosis. To date, research on the AM–AS herb pair has mainly focused on the chemical compositions associated with therapeutic efficacy. However, supermolecules actually exist in herb codecoctions, and their self-assembly mechanism remains unclear. In this study, supermolecules originating from AM–AS codoping reactions (AA-NPs) were first reported. The chemical compositions of AA-NPs showed a dynamic self-assembly process. AA-NPs with different decoction times had similar surface groups and amorphous states; however, the size distributions of these nanoparticles might be different. Taking the interaction between Z-ligustilide and astragaloside IV as an example to understand the self-assembly mechanism of AA-NPs, it was found that the complex could be formed with a molar ratio of 2:1. Later, AA-NPs were proven to be effective in the treatment of myocardial fibrosis both in vivo and in vitro, the in-depth mechanisms of which were related to the recovery of cardiac function, reduced collagen deposition, and inhibition of the endothelial-to-mesenchymal transition that occurred in the process of myocardial fibrosis. Thus, AA-NPs may be the chemical material basis of the molecular mechanism of the AM–AS decoction in treating isoproterenol-induced myocardial fibrosis. Taken together, this work provides a supramolecular strategy for revealing the interaction between effective chemical components in herb-pair decoctions.

## 1 Introduction

Traditional herbal medicine (THM) has been applied for thousands of years in Asia and surrounding areas, helping in the prevention and treatment of diseases.^1^ In clinical use, two or more types of THMs, named herb pairs, are combined to achieve optimal therapeutic effects, which reflects the compositional rule of THM prescriptions. Currently, several classical herb pairs possess excellent efficacy, as assessed from long-term clinical experience; however, the in-depth mechanism still needs to be explored. *Astragalus membranaceus* (Fisch.) *Bge.* (AM) and *Angelica sinensis* (Oliv.) *Diels.* (AS), a commonly used herb pair, has the effect of supplementing *qi* (vital energy) and nourishing blood (body circulation).^2^ The AM–AS herb pair has always been a research hotspot from the perspectives of chemical composition, substance metabolism, pharmacological effects, quality control, and clinical applications.^3^ More importantly, previous studies were mainly focused on the study of the *Danggui Buxue* decoction. The *Danggui Buxue* decoction, with an AM/AS weight ratio of 5:1, has been used for over 800 years for the treatment of *qi* and blood deficiency-related diseases.^4^ Myocardial fibrosis, belonging to the category of chest arthralgia in Chinese medicine theory, shows the basic features of *qi* and blood deficiency.^5^ The mechanism of pathological changes in the extracellular matrix of cardiomyocytes involves the activation of myocardial fibroblasts, contributing to the excessive accumulation of collagen fibers. Given the side effects of long-term use of statins, there is a need to develop safe and effective drugs with low toxicity to benefit patients with myocardial fibrosis.^6^ In recent years, the AM–AS codecoction has been used as a natural medicine to prevent and treat myocardial fibrosis.^7^ An understanding of the mechanism of the AM–AS codecoction in the treatment of myocardial fibrosis will further enrich the basic research of its pharmacodynamic substances.

Decoction is the preferred dosage form for treating complex diseases.^8^ Previously, studies on the pharmacological substance basis of decoctions were mostly focused on small molecular components.^9^ Nevertheless, it is often difficult to evaluate the overall characteristics of herb decoctions by adopting a single effective ingredient and imitating the research idea of chemical medicine. Modern research on herb decoctions should clarify the characteristics of each component and the interaction between them. Gradually, an increasing number of researchers have found that nanoscale particles are ubiquitous in decoctions, which are associated with their pharmacological effects.^10^ For example, a colloid system with a size of 162 nm isolated from the codecoction of *Glycyrrhiza uralensis* Fisch and *Coptis chinensis* Franch was composed of 34 flavonoids, 34 alkaloids, and 2 triterpenoids, showing stronger antibacterial activity than a noncolloidal solution.^1^ Similarly, the nanoscale aggregates originating from *Gegen Qinlian* decoction exhibited stronger protection of the viability and function of β-cells in vitro.^11^ The resulting data also indicated that aggregates and precipitates in herbal decoctions should be carefully handled in production and pharmacological research. In addition, nanoparticles discovered in *Huanglian Jiedu*, *Huanglian*, *Maxing Shigan*, *Baihu*, *Liujunzi*, and *Shaoyao Gancao* decoctions showed superior properties and activities.^10,12,13^ Even in Turkish gall extracts, researchers found the presence of spherical nanoparticles that possessed the ideal size, strong antioxidant activity in scavenging various radicals, and antibacterial performance.^14^ However, whether nanoparticles exist in the AM–AS codecoction remains unclear. Most of these nanoparticles with sizes in the range of 100–600 nm had strong stability and high bioavailability.^10^ Through ultrahigh-performance liquid chromatography high-resolution mass spectrometry (UHPLC-HR-MS) analysis, it was found that the chemical compositions of nanoparticles in decoctions were extremely complex, including saponins, polysaccharides, flavonoids, and alkaloids.^1^ Therefore, it is challenging to study the self-assembly process and mechanism of these nanoparticles formed from the chemical components.

In 1973, the concept of supramolecular chemistry was proposed, which mainly involves supramolecular aggregates formed by self-assembly of two or more molecules through noncovalent intermolecular bonding forces.^15^ As this concept gradually became well-known, researchers discovered many supramolecules in soups, such as fish soup, freshwater clam soup, and pork bone soup.^16^ Decocting is not a single process of extracting components from hot water, which drives the components to spontaneously form supramolecular aggregates with a certain particle size.^13^ The processing of food soups is similar to that of herb decoctions. Integrating the concept of supramolecular chemistry, nanoparticles in herb decoctions are supramolecules produced by the self-assembly of chemical components under the action of noncovalent forces, including hydrogen bonds, van der Waals forces, hydrophobic forces, and electrostatic attraction.^17^ In conclusion, herb decoctions could be considered carrier-free small-molecule coassembly platforms to design and construct supramolecules, which also provides a novel perspective for elucidating the pharmacodynamic material basis of decoctions.

Considering the saponins, flavonoids, and lactones contained in the AM–AS herb pair, we speculated that supermolecules might be formed spontaneously after the preparation of the codecoction. In this study, for the first time, supermolecules known as AA-NPs were separated from a codecoction of AM–AS in water by ultrafiltration–centrifugation. The effect of different decoction times on the formation of self-assembled structures was first investigated. The chemical compositions of AA-NPs showed a dynamic self-assembly process. AA-NPs with different decoction times had similar surface groups and amorphous states; however, the size distributions of these nanoparticles might be different. It is of great significance to determine an appropriate codecoction time to obtain nanoparticles with the ideal size. Combined with multiple technologies, the morphology, chemical compositions, optical properties, and stability were further evaluated. As a result, AA-NPs with irregular spherical shapes and negatively charged surfaces exhibited good stability and water solubility. 43 chemical components were identified in AA-NPs by UHPLC-HR-MS analysis, including 11 saponins, 13 flavonoids, 3 amino acids, 4 phthalides, 4 organic acids, and 8 others. All identified components were involved in the self-assembly process of AA-NPs. However, according to our previous results, Z-ligustilide (LIG) and astragaloside IV (AIV) showed the highest encapsulation efficiency and slowest release from AA-NPs. Herein, we attempt to elucidate the potential self-assembly mechanism by investigating the interactions between LIG and AIV contained in AA-NPs. As expected, various characterization technologies, such as scanning electron microscopy (SEM), ultraviolet–visible (UV–vis) spectroscopy, Fourier-transform infrared (FTIR) spectroscopy, UHPLC-HR-MS, and molecular dynamics (MD) simulation, were used to verify the interaction between LIG and AIV. A self-assembled LIG–AIV complex with a molar ratio of 2:1 was identified, the formation of which might be closely related to the hydrophobic part of LIG and the quaternary ring skeleton of AIV. Later, AA-NPs were proven to be effective in the treatment of isoproterenol-induced myocardial fibrosis in a mice model, the in-depth mechanisms of which were associated with restoring cardiac function, reducing collagen-I deposition, and inhibiting endothelial-to-mesenchymal transition (EndMT). Meanwhile, Ang II-induced human umbilical vein endothelial cell (HUVEC) and human microvascular endothelial cell (HMEC-1) models were established to confirm the inhibitory effect of AA-NPs on the EndMT process in vitro. In summary, this study considered AA-NPs as an example to explore the formation mechanism of supermolecules discovered in herb decoctions, contributing to designing carrier-free small-molecule nanoparticles and understanding the pharmacodynamic material basis of herb decoctions.

## 2 Results and Discussion

### 2.1 Effect of Different Codecoction Times on the AA-NP Structures

**Figure 1:**
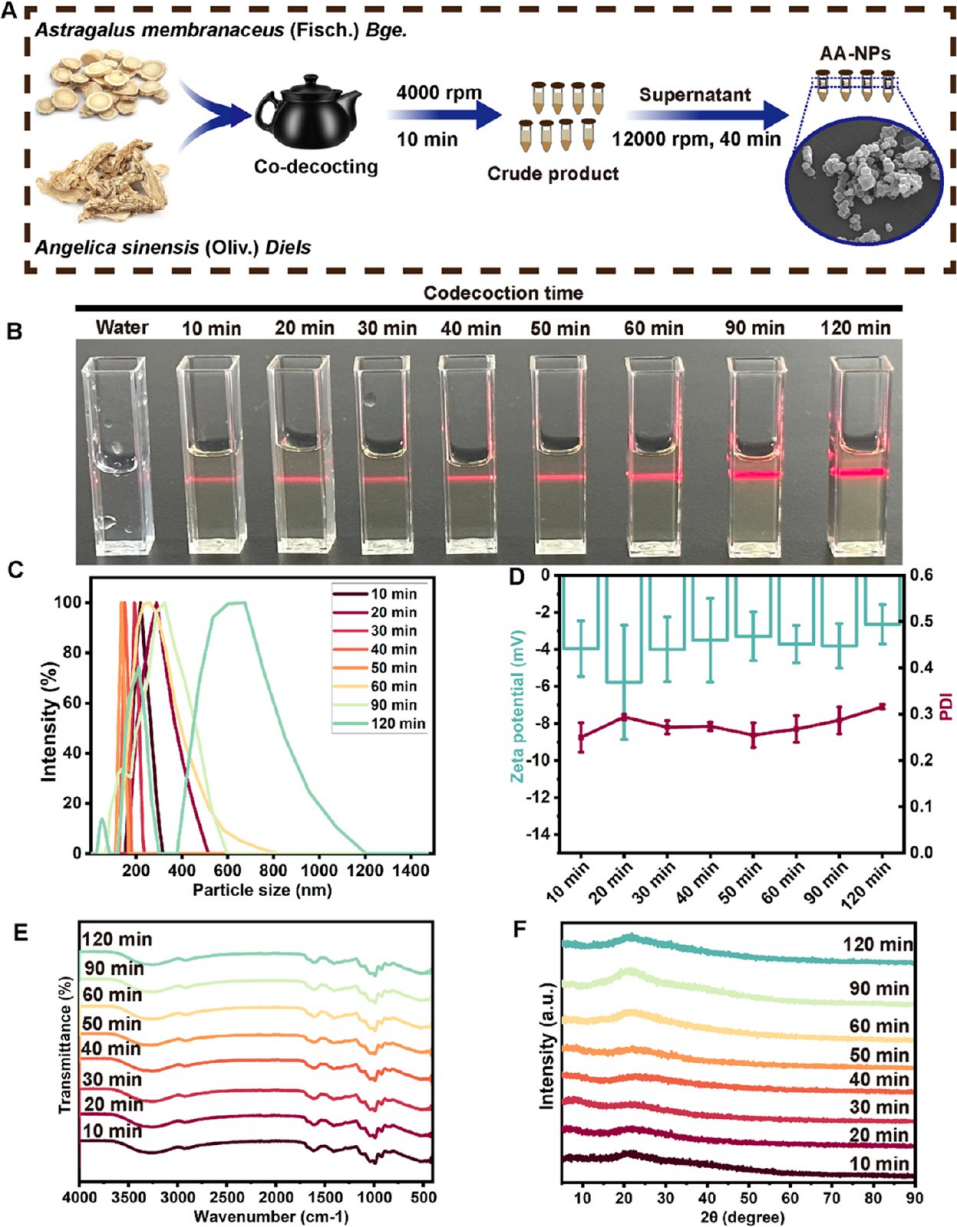
Exploration of the formation of the AA-NP structure with different codecoction times. (A) Schematic diagram showing the extraction AA-NPs from the AM–AS codecoction. (B) Tyndall effect of AA-NPs with different codecoction times. (C) Size distribution and (D) PDI and ζ-potential of AA-NPs by dynamic light scattering analysis. (E) FTIR spectra and (F) XRD spectra of AA-NPs with different codecoction times. All data are presented as the mean ± SD (*n* = 3).

The combination of AM and AS has been widely applied in the clinic. In this study, we explored the mechanism of compatibility of the AM–AS herb pair from the perspective of supramolecule formation. As shown in Figure 1A, the ultrafiltration–centrifugation method was applied for the extraction of AA-NPs from the AM–AS codecoction, which could better maintain the original state of the nanoparticles. AA-NPs with different codecoction times presented a transparent yellowish solution in room light and showed an obvious Tyndall effect after laser irradiation, which further verified the existence of nanoparticles in the whole preparation process of the codecoction (Figure 1B). DLS analysis revealed that the size distribution of AA-NPs decreased from 196.53 ± 8.6 to 133.66 ± 7.49 nm as the codecoction time increased from 10 to 50 min (Figure 1C). On prolonging the codecoction time to 60, 90, and 120 min, the particle size of AA-NPs gradually changed to 188.93 ± 2.18, 254.52 ± 8.95, and 279.77 ± 3.74 nm, respectively. Therefore, the size of AA-NPs decreased first and then increased during a codecoction time of 120 min, suggesting that the chemical composition of AA-NPs exhibited a dynamic self-assembly process. The particle dispersion of AA-NPs with different codecoction times also showed a similar variation trend (Figure 1D). After a codecoction time of over 90 min, PDI values of AA-NPs were greater than 0.3, indicating that the colloidal system gradually became unstable. Interestingly, there was no significant difference in the ζ-potential values of AA-NPs during the whole codecoction preparation process, which might be closely related to the surface functional groups of AA-NPs. Further, FTIR spectroscopy was performed to explore the surface functional groups of AA-NPs. As illustrated in Figure 1E, the broad absorption peak of AA-NPs at 3259 cm^–1^ was attributed to the stretching vibration of the O–H or N–H bonds.^18^ Two absorption peaks at 2922 and 1408 cm^–1^ corresponded to the tensile vibration and in-plane bending vibration of sp^3^ C–H, respectively.^19^ Furthermore, peaks at 1612 and 1269 cm^–1^ were attributed to the stretching vibrations of C=O and C–O from the carboxylic acid group, respectively.^20^ Peaks at 985, 922, and 830 cm^–1^ indicated the stretching vibration of C–O–C, variable angle vibration of O–H, and variable angle vibration of sp^3^ C–H, respectively. As a result, the surface of AA-NPs was rich in hydroxyl, amino, and carboxyl groups, helping to maintain stability during the codecoction process. Meanwhile, the absorption peak shift observed in the FTIR spectra was negligible, which confirmed that AA-NPs with different codecoction times had similar surface functional groups. This result further explained that the surface potential values of AA-NPs obtained with different codecoction times were almost the same. Besides, Figure 1F shows that the XRD spectra of AA-NPs with different codecoction times exhibited similar broad peaks at 21.62°. This phenomenon could be attributed to the amorphous state of AA-NPs, which is consistent with their excellent aqueous solubility.^21^ AA-NPs extracted from the AM–AS codecoction had similar surface groups and amorphous states at different codecoction times; however, the size distributions of these nanoparticles might be different. The codecoction process is the earliest and most common processing method of herbs, which drives the chemical components of herbs to migrate into the decoction to form self-assembled nanostructures.^22^ Consistent with previous reports, different codecoction times affect the particle size of the self-assemblies generated from the codecoction of *Coptidis rhizoma* and *Scutellariae Radix* and consequently their antibacterial effect.^23^ Nanoparticles separated from the *Baihu* decoction, with a size of around 100 nm, were easily absorbed by cells and showed antipyretic effects.^24^ It is of great significance to determine the appropriate codecoction time to obtain nanoparticles with the ideal size. In this study, the smallest particle size (133.66 ± 7.49 nm) and narrowest size distribution (PDI: 0.255 ± 0.027) of AA-NPs were observed after a codecoction time of 50 min. The above evidence confirmed the successful separation of nanoparticles in the AM–AS codecoction, and their properties need to be further characterized.

### 2.2 Characterization of AA-NPs

**Figure 2:**
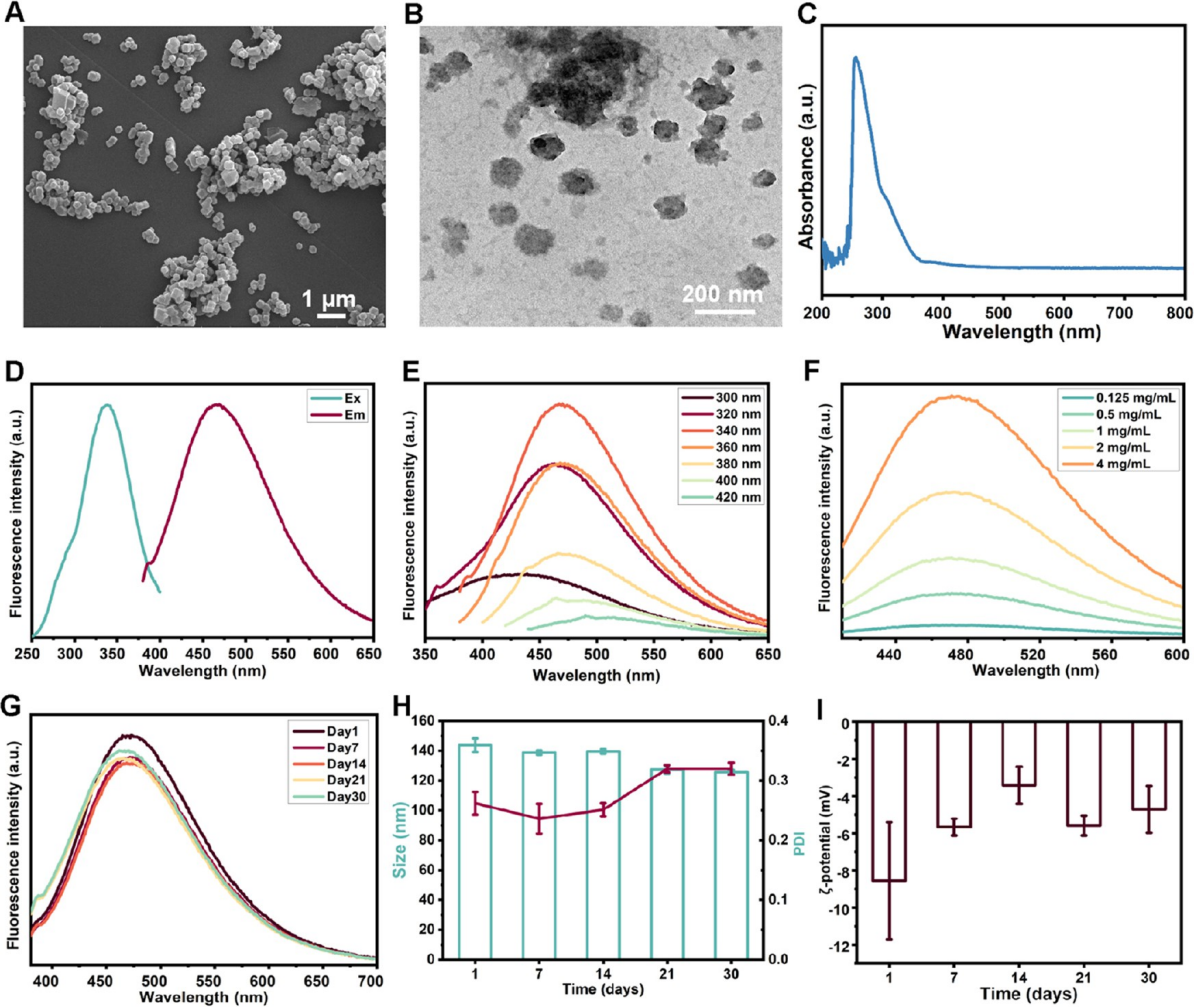
Characterization of AA-NPs. (A) SEM and (B) TEM images of AA-NPs. (C) UV–vis absorption spectrum of AA-NPs. (D) Fluorescence excitation and emission spectra of AA-NPs. (E) Fluorescence emission spectra of AA-NPs with excitation wavelengths of 300–420 nm. (F) Changes in the fluorescence intensity of AA-NPs at different concentrations. (G) Fluorescence stability of AA-NPs within 30 days. (H) Particle size and PDI and (I) ζ-potential of AA-NPs during the 30 day storage period. All data are presented as the mean ± SD (*n* = 3).

Experimentally, AA-NPs were successfully obtained using a one-step strategy through the preparation of a codecoction of AM–AS in water followed by ultrafiltration–centrifugation. The representative SEM image indicated that the AA-NPs exhibited a polygonal shape with a size of 150 nm (Figure 2A). Further, the irregular spherical shape of AA-NPs was also observed in the TEM image (Figure 2B). In the UV–vis absorption spectrum, a broad peak at 256 nm of AA-NPs was attributed to the π–π* transition of carbon, suggesting the presence of the benzene ring or C=C (Figure 2C).^10^ Interestingly, AA-NPs also possessed a certain fluorescence. The fluorescence spectrum results are shown in Figure 2D. The optimal excitation wavelength and emission wavelength were 340 and 470 nm, respectively. In addition, AA-NPs exhibited excitation-dependent emission properties (Figure 2E). The fluorescence intensity increased with increasing concentration of AA-NPs (Figure 2F), revealing that the luminescent groups were derived from nanoparticles rather than chemical components. Considering the subsequent in vivo application, the storage stability of the AA-NPs at −80 °C for 30 days was determined. Compared with day 1, the fluorescence intensity of AA-NPs showed a certain decrease on day 7; however, there was no significant change in the fluorescence intensity from days 7 to 30, demonstrating good fluorescence stability (Figure 2G). Later, both the particle size and ζ-potential of AA-NPs changed negligibly during 30 days of storage (Figure 2H,I). However, the PDI of AA-NPs on days 21 and 30 exceeded 0.3, which may be ascribed to the weakened redispersibility in water and the presence of large particles. In general, these results illustrated that the surface functional groups and amorphous state of AA-NPs contributed to their special fluorescence properties and excellent storage stability. The chemical compositions of AA-NPs and their interactions are not fully understood.

### 2.3 Chemical Compositions of AA-NPs

**Figure 3:**
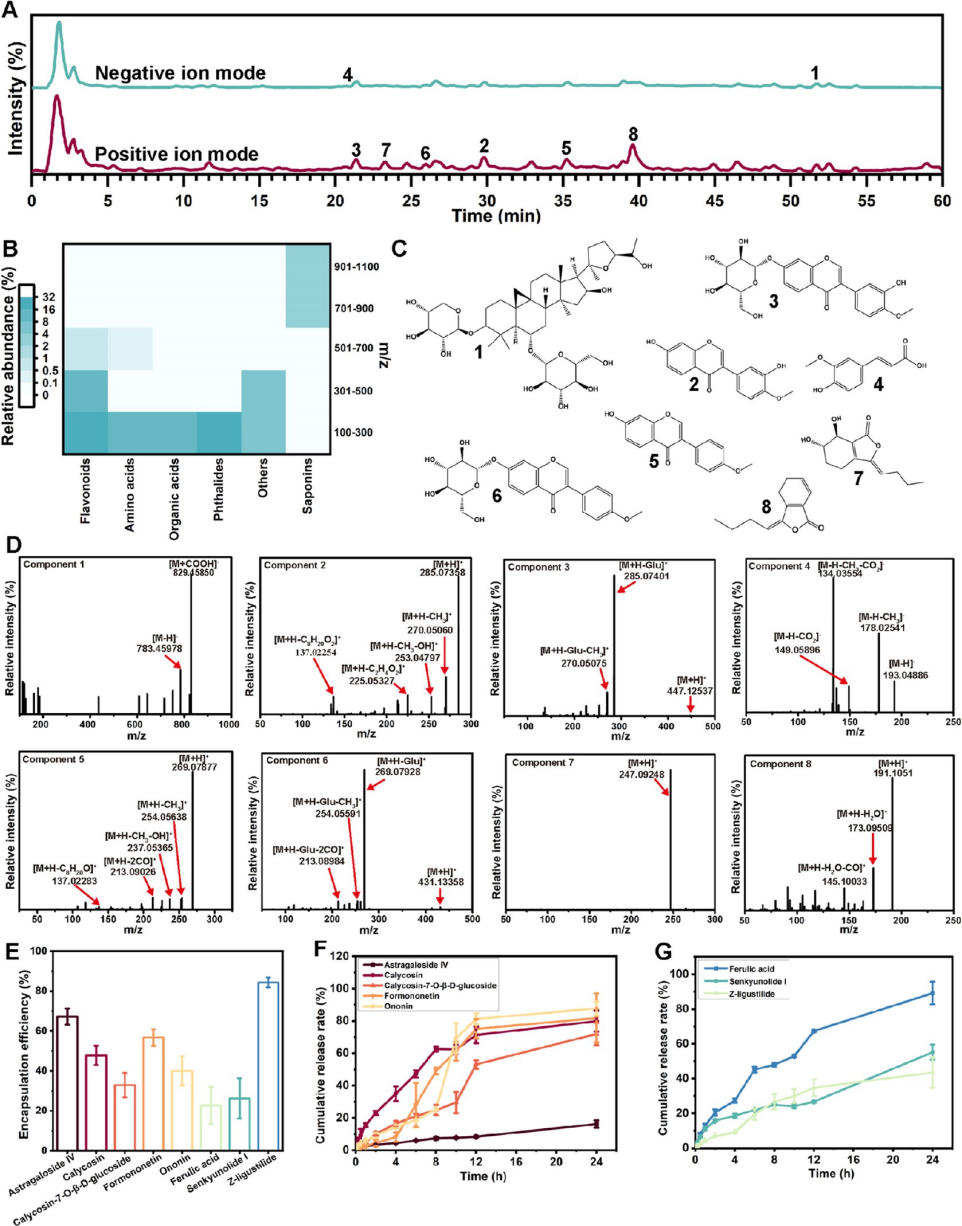
Identification of the chemical composition of AA-NPs. (A) UHPLC-HR-MS analysis of AA-NPs in positive and negative ion modes. Peaks 1–8 present the representative components of AA-NPs: 1, astragaloside IV; 2, calycosin; 3, calycosin-7-*O*-β-d-glucoside; 4, ferulic acid; 5, formononetin; 6, ononin; 7, senkyunolide I; and 8, Z-ligustilide. (B) Heatmap visualizing the relative abundances of the identified components in AA-NPs. (C) Chemical structures of components 1–8 of AA-NPs. (D) Cleavage fragment ions of components 1–8 of AA-NPs. (E) Encapsulation efficiency of the five components in AM and the three components in AS of AA-NPs. Cumulative release rate of the (F) five components in AM and (G) three components in AS of AA-NPs. All data are presented as the mean ± SD (*n* = 3).

Chemical compositions of AA-NPs were detected by UHPLC-HR-MS in positive and negative ion modes, and the scan range was *m*/*z* 100–1500 Da (Figure 3A). We systematically analyzed the retention time, parent ion, and fragment ion of all components. In total, 43 components were tentatively identified in AA-NPs, the detailed information on which is presented in Table S1. The main components of AA-NPs included 11 saponins, 13 flavonoids, 3 amino acids, 4 phthalides, 4 organic acids, and 8 others. Among them, the peak positions of eight active components are marked in Figure 3A, including astragaloside IV (1), calycosin (2), calycosin-7-*O*-β-d-glucoside (3), ferulic acid (4), formononetin (5), ononin (6), senkyunolide I (7), and Z-ligustilide (8). As shown in Table S1, the peak areas of the identified components in AA-NPs were semiquantified. The relative abundance of 43 identified components in AA-NPs was visualized in a heatmap (Figure 3B). As a result, the relative abundances of the components with *m*/*z* values of 100–300 and 301–500 in AA-NPs were 75.75 and 15.21%, respectively, mainly consisting of flavonoids, amino acids, organic acids, and phthalides. Besides, saponins were mainly distributed at *m*/*z* values above 700. The chemical structures represented by peaks 1–8 are illustrated in Figure 3C.

Then, the fragmentation pathways of the representative components were investigated. For example, as shown in Figure 3D, component 1 showed [M + COOH]^−^ at *m*/*z* 829.45850 and [M – H]^−^ at *m*/*z* 783.45978 in the negative ion mode, which could be identified as astragaloside IV. Astragaloside IV, a cycloartane tetracyclic triterpene saponin in AM, is the quality control marker recorded by the Chinese Pharmacopoeia (2020 edition).^1^ Flavonoids, with 2-phenylchromone as the framework, are another major bioactive component of AM.^7^ Thirteen flavonoids were found in AA-NPs, such as calycosin, calycosin-7-*O*-β-d-glucoside, formononetin, and ononin, possessing similar fragmentation pathways. Components 2 and 3 were proposed to be calycosin and calycosin-7-*O*-β-d-glucoside, respectively. In the positive ion mode, the protonated molecule [M + H]^+^ of component 2 was located at *m*/*z* 285.07358. The major fragment ions of [M + H–CH_3_]^+^ at *m*/*z* 270.05060, [M + H–CH_3_–OH]^+^ at *m*/*z* 253.04797, and [M + H–CH_3_–OH–CO]^+^ at *m*/*z* 225.05327 were attributed to the continuous loss of a CH_3_ moiety, an OH moiety, and a CO moiety, respectively. The fragment ion of *m*/*z* 137.02254 was produced by the retro Diels–Alder reaction from that at *m*/*z* 285.07358 ([M + H]^+^).^1^ Similarly, component 3 produced a precursor ion at *m*/*z* 447.12537 ([M + H]^+^), yielding the component 2 fragment ion at *m*/*z* 285.07401 ([M + H-Glu]^+^) by breaking the glycosidic bond and then undergoing a pyrolysis process similar to that of component 2. Therefore, *m*/*z* 285.07358, 270.05060, 253.04797, and 137.02254 could be considered diagnostic ions of the calycosin parent nucleus. Furthermore, components 5 and 6 were identified as formononetin and ononin, respectively. Specifically, the fragment ions at *m*/*z* 254.05591 ([M + H–CH_3_]^+^) and *m*/*z* 237.05365 ([M + H–CH_3_–OH]^+^) were formed through the loss of a CH_3_ moiety and subsequent loss of an OH moiety from *m*/*z* 269.07877 ([M + H]^+^). The fragment ions of *m*/*z* 213.09026 ([M + H-2CO]^+^) and 137.02283 ([M + H–C_8_H_20_O]^+^) were produced by the loss of 2CO and the retro Diels–Alder reaction from *m*/*z* 269.07877 ([M + H]^+^), respectively. Likewise, the precursor ion of *m*/*z* at 431.13358 ([M + H]^+^) removed a glucose fragment and formed the strongest fragment ion at *m*/*z* 269.07928 ([M + H-Glu]^+^). Therefore, *m*/*z* 269.07877, 254.05591, and 137.02283 could be considered diagnostic ions of the formononetin parent nucleus. Component 4 (ferulic acid), an organic acid, is the major component of AS. The deprotonated molecule of ferulic acid was observed at *m*/*z* 193.04886 ([M – H]^−^). The major fragment ions of [M – H–CH_3_]^−^ at *m*/*z* 178.02541 and [M – H–CH_3_–CO_2_]^−^ at *m*/*z* 134.03554 were attributed to the continuous loss of a CH_3_ moiety and a CO_2_ moiety. In addition, a fragment ion at *m*/*z* 149.05896 was also formed because of the elimination of a CO_2_ moiety through the parent ion at *m*/*z* 193.04886 ([M – H]^−^). Components 7 and 8 were identified as senkyunolide I and Z-ligustilide, respectively. Component 8 (Z-ligustilide) exhibited a positive ion at *m*/*z* 191.1051 and possessed the fragment ions of [M + H–H_2_O]^+^ (*m*/*z* 173.09509) and [M + H–H_2_O–CO]^+^ (*m*/*z* 145.10033).

The encapsulation and release of eight components in AA-NPs were further investigated using ultrafiltration–centrifugation and dialysis methods, respectively. The encapsulation efficiencies of astragaloside IV, calycosin, calycosin-7-*O*-β-d-glucoside, formononetin, and ononin in AA-NPs were 67.22 ± 4.08, 47.83 ± 4.83, 32.92 ± 6.11, 57.77 ± 4.15, and 40.01 ± 7.32%, respectively (Figure 3E). Meanwhile, the encapsulation efficiencies of ferulic acid, senkyunolide I, and Z-ligustilide were 22.71 ± 9.27, 26.22 ± 10.03, and 84.30 ± 2.52%, respectively (Figure 3E). Importantly, the top two components with the highest encapsulation efficiency were astragaloside IV and Z-ligustilide, suggesting that these two components might play a vital role in the self-assembly process of AA-NPs. In Figure 3F,3G, calycosin, calycosin-7-*O*-β-d-glucoside, formononetin, ononin, and ferulic acid showed similar release properties, with a 24 h release rate of approximately 80%, indicating effective encapsulation and sustained release. However, the 24 h release rates of astragaloside IV and Z-ligustilide did not exceed 50%. This phenomenon might be attributed to the presence of interactions between these two components that block the release. We observed that the structure of astragaloside IV contained hydrogen bond donors and receptors, as well as a hydrophobic skeleton, providing the driving forces for self-assembly.^25^ Thus, taking these two components as examples to explore the self-assembly mechanism of AA-NPs may be feasible.

### 2.4 Analysis of the Interaction between LIG and AIV

**Figure 4:**
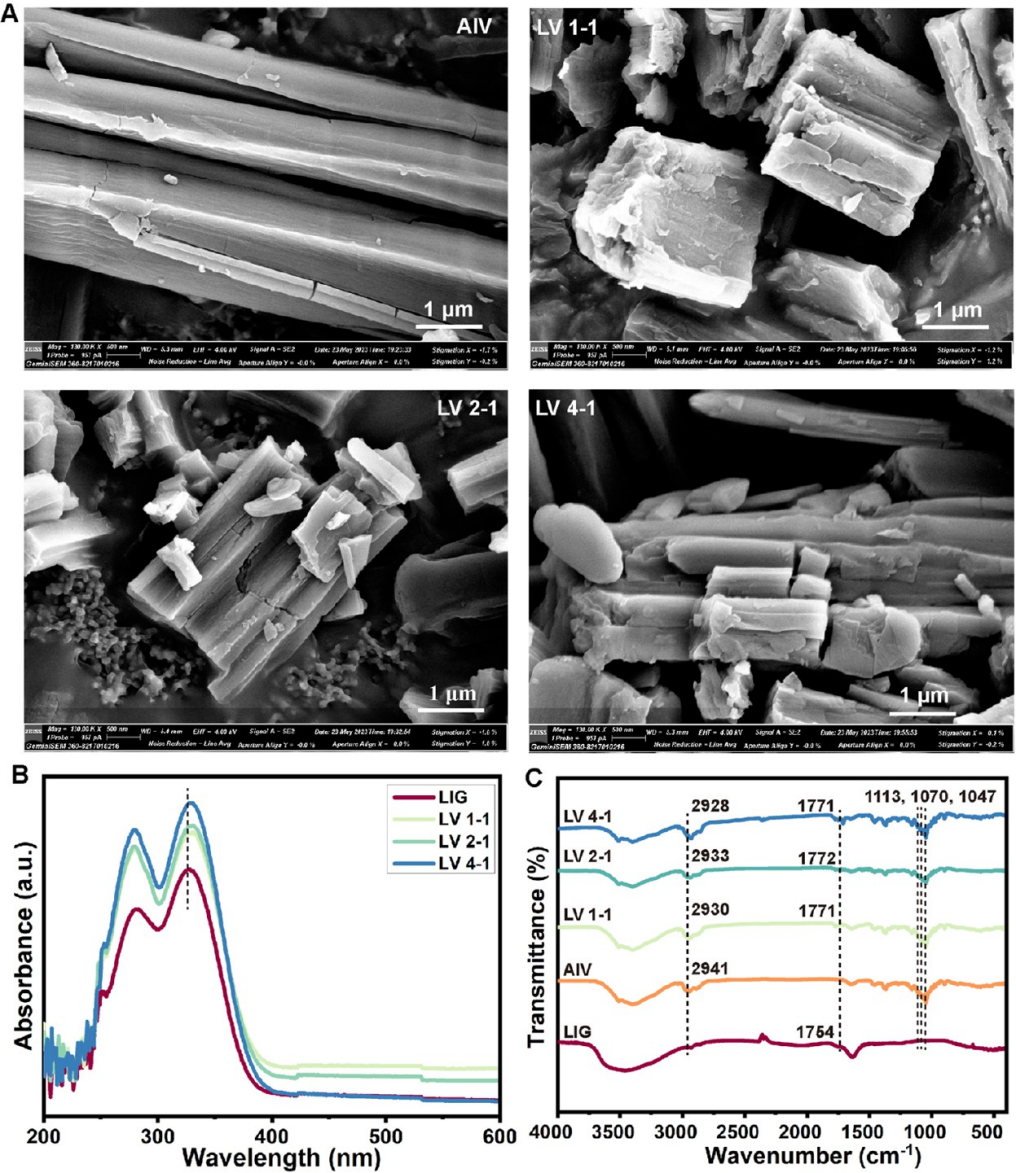
Self-assembly of LIG and AIV. (A) SEM images of AIV, LV 1–1, LV 2–1, and LV 4–1. (B) UV–vis absorption spectra and (C) FTIR spectra of AIV, LIG, LV 1–1, LV 2–1, and LV 4–1.

Based on the results of the UHPLC-HR-MS analysis, AA-NPs were mainly composed of saponins, flavonoids, and lactones. More importantly, the release of lactones and saponins in AA-NPs was much lower than those in other types of components. From this, we speculated that there may be interactions between LIG and AIV, which, in turn, affect their release. To evaluate the self-assembly mechanism of AA-NPs, the interaction between LIG and AIV was analyzed by simple ultrasonic dispersion in the aqueous phase. As shown in Figure 4A, the micromorphology of LIG–AIV self-assembly complexes with different molar ratios was characterized by SEM images. AIV had a complete, smooth, and slender fibrous structure. After adding LIG into the system, compared to pure AIV, the morphology of the self-assembled complex became rough and fragmented, similar to that in a previous study.^26^ There was no significant morphological change between the different molar ratios of LIG and AIV. It could be inferred that there were interactions between LIG and AIV that changed the micromorphology of the complex. However, the underlying mechanism of the interactions between LIG and AIV is still unclear. Therefore, spectral technologies such as UV–vis, fluorescence, and FTIR spectroscopies were used to identify the interaction forces between LIG and AIV. According to Figure 4B, the absorption peak around 280 nm in the UV–vis spectra belonged to the π–π* transition of the aromatic ring of LIG. In addition, another peak at 326 nm was attributed to the characteristic absorption of carbonyl groups of LIG. Compared with LIG, the carbonyl groups of LV 1–1, LV 2–1, and LV 4–1 showed obvious red-shifts. This result revealed that the steric hindrance of AIV molecules interfered with the conjugated system of LIG, which confirmed the existence of the hydrophobic interactions between LIG and AIV.^25^ Furthermore, the fluorescence spectra of LIG, LV 1–1, LV 2–1, and LV 4–1 were obtained to understand the intermolecular interactions (Figure S1). Compared with LIG, the fluorescence intensities of LV 1–1, LV 2–1, and LV 4–1 were generally decreased, indicating that AIV could weaken the fluorescence intensity of LIG. This result could be explained by the enhanced hydrophobicity of AIV, which further validated the hydrophobic interaction between LIG and AIV.^26^ In the FTIR spectra, compared with AIV (2941 cm^–1^), the C–H absorption peaks of LV 1–1, LV 2–1, and LV 4–1 were present at 2930, 2933, and 2928 cm^–1^, respectively, showing the certain blue-shifts (Figure 4C). These specific band changes suggested that hydrophobic interactions were the driving force for the interaction between LIG and AIV, which is consistent with the UV–vis and fluorescence spectral results. In addition, the peak appearing at 1754 cm^–1^ represents the stretching vibrations of the C=O bond of LIG. When LIG was combined with AIV, red-shifts from 1754 to 1771, 1772, and 1771 cm^–1^ were observed in LV 1–1, LV 2–1, and LV 4–1, respectively. The change of the C=O vibration peak shift further indicates that the hydrophobic effect promotes the formation of the LIG–AIV complex.^26^ As shown in Figure 4C, the three peaks at 1113, 1070, and 1047 cm^–1^ were the characteristic peaks of hydroxyl groups on the glycosyl group of AIV. There was no significant absorption peak shift after the addition of LIG, indicating that the interaction site of LIG and AIV was not at the two glycosyl groups of AIV. Taken together, the above analysis revealed that hydrophobic interactions might be the main driving force for the formation of the LIG–AIV complex. However, the binding ratio and sites between LIG and AIV molecules still need to be further explored.

### 2.5 Identification of the Self-Assembled LIG–AIV Complex

**Figure 5:**
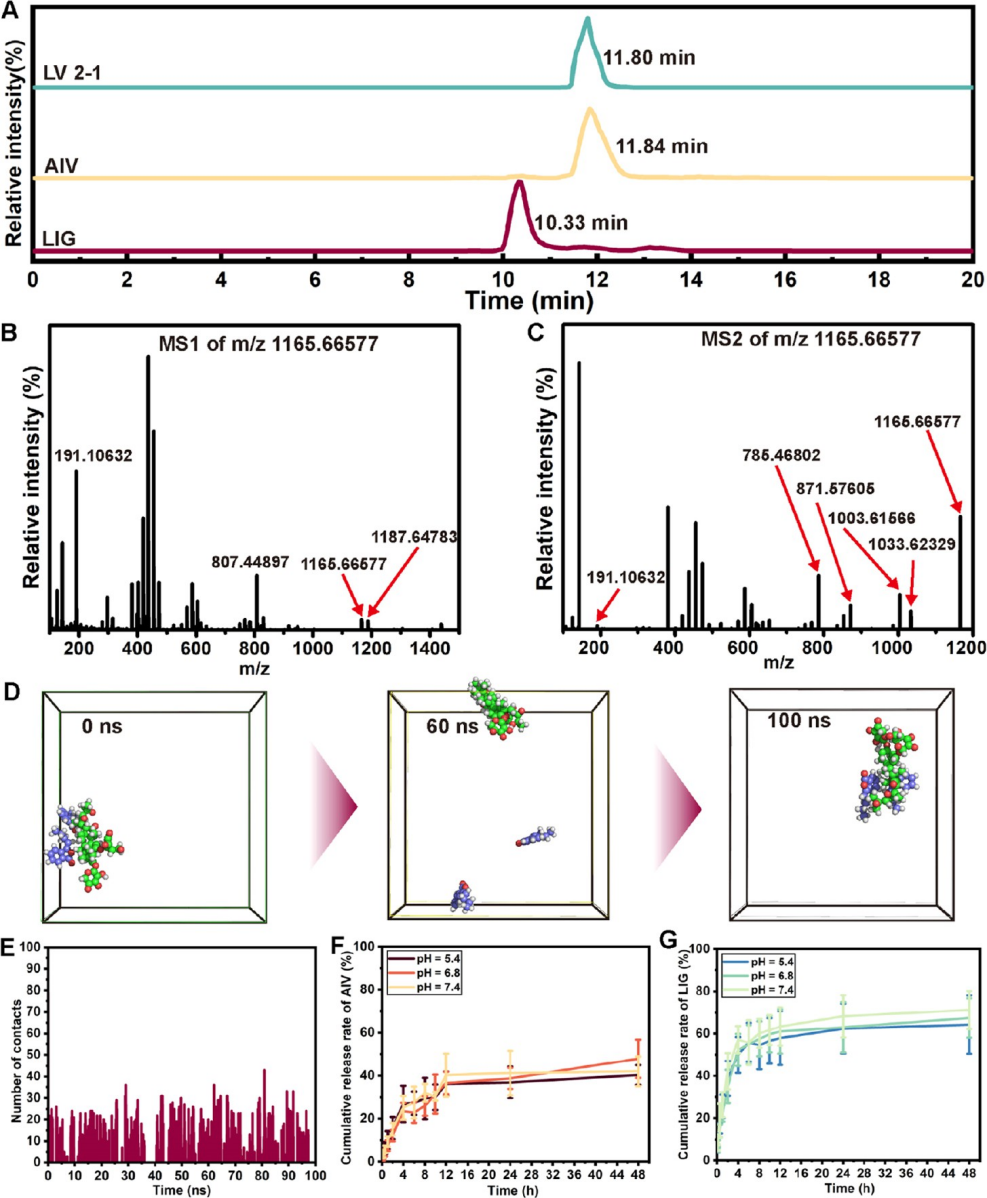
Identification of the self-assembled LIG–AIV complex. (A) UHPLC-HR-MS analysis of LIG, AIV, and LV 2–1 in positive ion mode. (B) Mass spectrometry characterization and (C) cleavage fragment ions of LV 2–1. (D) Spatial structural changes in the LIG–AIV system at 0, 60, and 100 ns during the molecular dynamic simulation. (E) Number of contact points for one AIV molecule binding with two LIG molecules over simulation for 100 ns. Release kinetics of (F) AIV and (G) LIG from LV 2–1 at pH = 5.4, 6.8, and 7.4, respectively. All data are presented as the mean ± SD (*n* = 3).

It was encouraging that the self-assembled LIG–AIV complex was detected by UHPLC-HR-MS analysis. As shown in Figure 5A, the retention times of LIG, AIV, and LV 2–1 were 10.33, 11.84, and 11.80 min, respectively. Furthermore, the results of Figure 5B confirmed that the self-assembled LIG–AIV complex was detected under positive ion mode at *m*/*z* 1165.66577 [M + H]^+^ and 1187.64783 [M + Na]^+^, with a binding ratio of 2:1. Specifically, two fragment ions at *m*/*z* 1033.62329 ([M + H–C_5_H_8_O_4_]^+^) and *m*/*z* 1003.61566 ([M + H–C_6_H_10_O_5_]^+^) were formed through the loss of a glycosyl group at one site (Figure 5C). Subsequently, the fragment ion at *m*/*z* 871.57202 ([M + H–C_5_H_8_O_4_–C_6_H_10_O_5_]^+^) was generated after the loss of two glycosyl groups. Furthermore, the fragment ion of [M + H-2C_12_H_14_O_2_]^+^ at *m*/*z* 785.46783 was attributed to the continuous loss of two molecules of LIG. Therefore, UHPLC-HR-MS analysis verified that the binding sites of the two LIG molecules were not on the glycosyl groups of AIV, which is consistent with the FTIR spectra.

Based on existing data, it is speculated that hydrophobic interactions could be the driving force for the self-assembly of LIG and AIV. To thoroughly investigate the interaction mechanism between these two small molecules, molecular dynamics simulations were conducted by using Amber 20 software. As shown in Figure 5D, to clearly visualize the dynamic process of continuous self-assembly of LIG and AIV, the spatial structural changes of the LIG–AIV system were recorded at 0, 60, and 100 ns. Finally, after 100 ns of simulation, an aggregation of LIG and AIV with a molar ratio of 2:1 was observed in the box system. Meanwhile, according to three-dimensional configuration analysis, LIG and AIV had significant hydrophobic interactions to maintain the stability of the spatial conformation (Figure S2). We also found a strong hydrophobic interaction between the hydrophobic part of LIG and the tetracyclic skeleton of the AIV structure.^25^ In Figure 5E, the number of contact points for one AIV molecule binding with two LIG molecules was up to 50 during the whole simulation process. Then, the release kinetics of the self-assembled complex (LV 2–1) at different pH values were investigated. As illustrated in Figure 5F, the cumulative release rate of AIV from LV 2–1 at pH values of 5.4, 6.8, and 7.4 reached approximately 40.34 ± 4.53, 47.84 ± 8.87, and 42.03 ± 6.88% within 48 h, respectively, showing the similar release activity. Meanwhile, the release profiles of LIG from LV 2–1 also presented the characteristics of rapid release within 12 h and sustained release between 12 and 48 h (Figure 5G). The sustained release profiles of AIV and LIG from LV 2–1 further confirmed the interactions between these two molecules. Notably, the cumulative release rate of LIG was higher than that of AIV, which might be related to the strong hydrophobicity of AIV resulting in the adhesion of the dialysis bag. Overall, the results of UHPLC-HR-MS analysis and molecular dynamics simulations suggested that LIG and AIV could stably bind with a molar ratio of 2:1 through hydrophobic interactions. In this study, we considered the LIG–AIV interaction process as a representative example to explore the self-assembly mechanism of AA-NPs. Nevertheless, the actual self-assembly process of AA-NPs might be much more complex and difficult to simulate.

### 2.6 AA-NPs Alleviated Isoprenaline-Induced Myocardial Fibrosis

**Figure 6:**
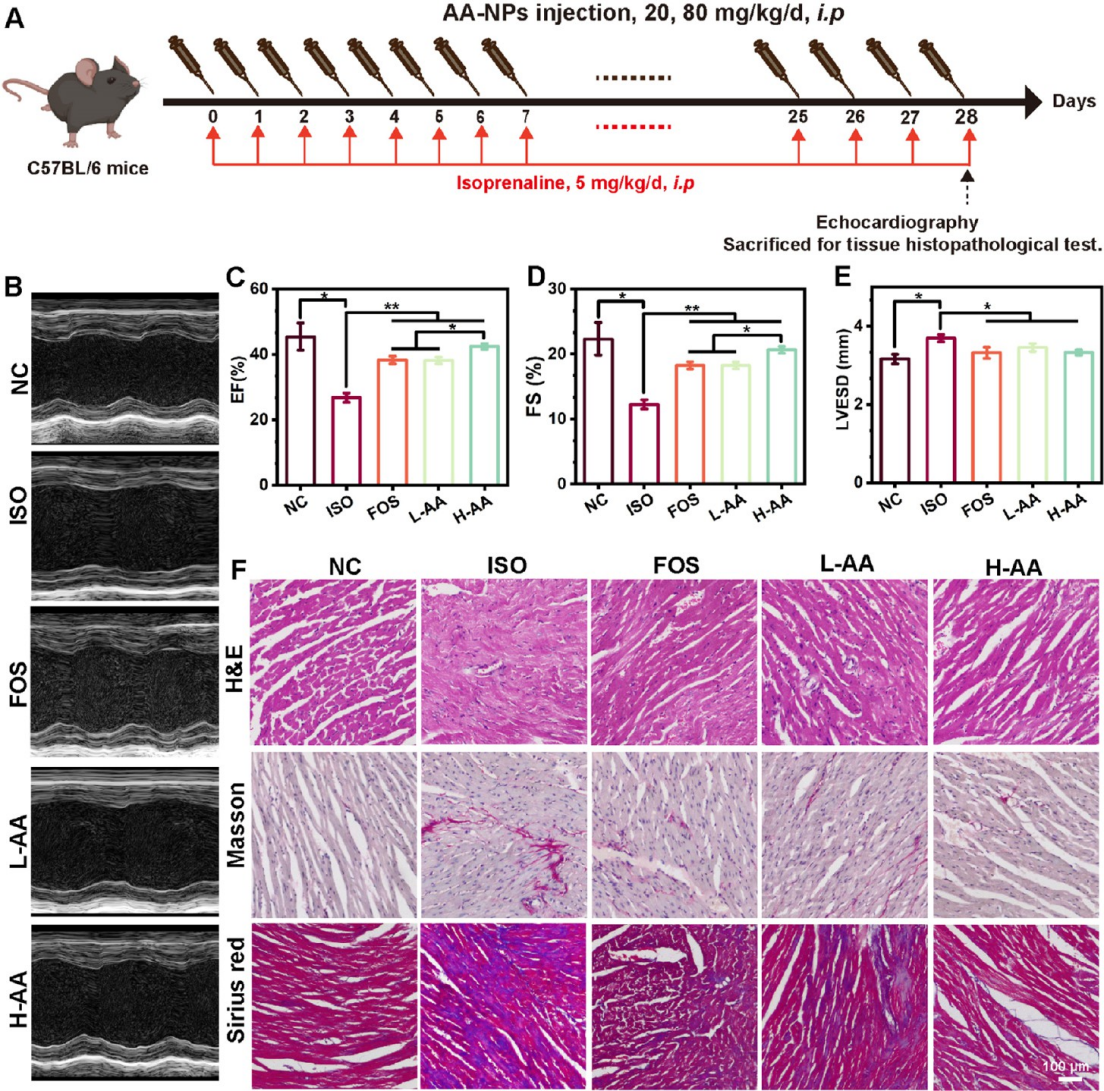
AA-NPs effectively inhibited the progression of isoprenaline-induced myocardial fibrosis. (A) Illustration of the construction process of the myocardial fibrosis C57BL/6 mouse model and AA-NPs therapy. (B) Representative graphs of echocardiography of the NC, ISO, FOS, L-AA, and H-AA groups. Quantitative evaluation of (C) EF%, (D) FS%, and (E) LVESD (mm) using echocardiography to assess impaired cardiac function. (F) H&E, Sirius red, and Masson staining of myocardial tissue sections. All data are presented as the mean ± SD (*n* = 3). * and ** represent *p* < 0.05 and *p* < 0.01, respectively.

Myocardial fibrosis is a common pathological process of several cardiovascular diseases, which is often accompanied by decreased cardiac function.^6^ Previous reports have confirmed that the AM–AS herb pair has a significant effect on the treatment of myocardial fibrosis.^7^ To verify the therapeutic efficacy of AA-NPs isolated from the AM–AS codecoction, isoprenaline-induced myocardial fibrosis was established and applied. Figure 6A illustrates the whole modeling and treatment process in this study. As shown in Figure 6B, the cardiac function was investigated via echocardiography, confirming the successful establishment of a myocardial fibrosis model. Specifically, mice with isoprenaline-induced myocardial fibrosis showed a significant decrease in cardiac function, manifested as a decrease in EF% and FS% and an increase in LVESD (Figure 6C–6E). However, after 28 days of AA-NP treatment, an increase in EF% and FS% and a decrease in LVESD were detected compared with the ISO group, suggesting the recovery of cardiac function. Furthermore, H&E staining showed significant myocardial damage in the ISO group, while AA-NPs had a protective effect on the injured myocardium (Figure 6F). As abnormal markers of myocardial fibrosis were caused by isoprenaline treatment, Sirius red staining and Masson staining were applied to visualize the fibrotic area. As shown in Figure 6F, compared to the ISO group, there was an obviously decreased fibrotic area with neatly arranged collagen after AA-NP treatment, indicating that AA-NPs could relieve isoprenaline-induced myocardial fibrosis.

### 2.7 AA-NPs Inhibited the EndMT Process to Ameliorate Myocardial Fibrosis In Vivo

**Figure 7:**
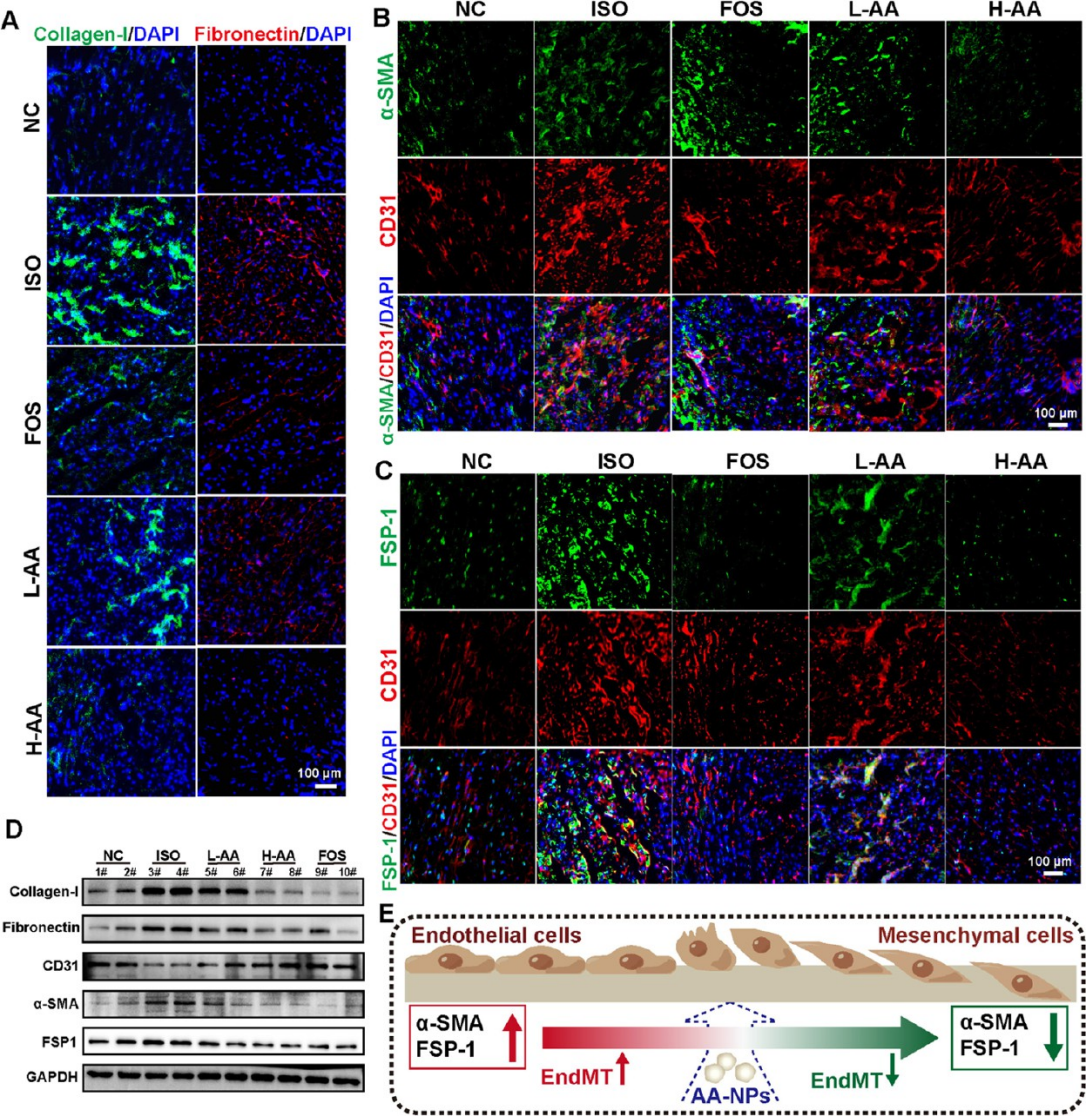
AA-NPs ameliorated isoprenaline-induced myocardial fibrosis by inhibiting EndMT. (A) Immunofluorescence staining of collagen-I (green) and fibronectin (red) in myocardial tissue sections. (B) Costaining of myocardial tissue sections for the vascular endothelial marker CD31 (red) and the mesenchymal marker α-SMA (green). (C) Costaining of myocardial tissue sections for the vascular endothelial marker CD31 (red) and the mesenchymal marker FSP-1 (green). (D) Expression levels of collagen-I, fibronectin, CD31, α-SMA, and FSP-1 proteins in the myocardial tissue using Western blot analysis. 1#–10# represent the myocardial tissue samples of 10 mice. (E) Illustration of AA-NPs interfering with the EndMT process. All data are presented as the mean ± SD (*n* = 3).

The above results have proved that AA-NPs could significantly restore cardiac function and reduce the fibrotic area in mice with myocardial fibrosis. However, the underlying mechanisms are still unclear. During the fibrosis process, excessive accumulation of collagen was caused by cardiac fibroblasts.^27^ Therefore, the expression levels of collagen-I and fibronectin were detected using immunofluorescence staining to investigate the effect of AA-NPs on the fibrosis process. As shown in Figure 7A, the overexpression of collagen-I and fibronectin was observed in the ISO group, indicating severe fibrosis. Compared with the ISO group, both L-AA and H-AA groups exhibited decreased expressions of collagen-I and fibronectin, further demonstrating that AA-NPs could inhibit collagen deposition and fiber adhesion in the process of fibrosis. Meanwhile, we also found that the expression levels of collagen-I and fibronectin in mice treated with high-dose AA-NPs (H-AA group) were lower than those in mice treated with fosinopril sodium (FOS group), suggesting that the therapeutic effect of AA-NPs on myocardial fibrosis was superior to that of conventional treatment.

Endothelial dysfunction plays a vital role in the occurrence and progression of cardiovascular diseases, one consequence of which is the occurrence of endothelial-to-mesenchymal transition (EndMT).^28^ EndMT, a new feature of pathological angiogenesis, contributes to the early development of myocardial fibrosis.^29^ In fact, endothelial cells undergo mesenchymal transition when they lose endothelial markers, such as cluster of differentiation 31 (CD31), to form mesenchymal phenotypes expressing α smooth muscle actin (α-SMA) and fibroblast-specific protein 1 (FSP-1).^30^ In this study, immunofluorescence analysis of endothelial marker CD31 revealed a significant increase in angiogenesis in ISO-induced cardiac fibrosis (Figure 7B). Colocalization of the endothelial marker CD31 and mesenchymal markers α-SMA and FSP-1 in the ISO group mice revealed that the proportion of endothelial cells with EndMT occurrence was obviously increased in them compared with the controls (Figure 7B,C), suggesting strong pathological angiogenesis. Compared with the ISO group, downregulated expressions of α-SMA and FSP-1 were observed in the vascular endothelium after 28 days of AA-NPs treatment. Especially, AA-NPs also showed a superior effect in reducing the EndMT process compared to conventional treatment, which is consistent with previous results. Then, the expression levels of collagen-I, fibronectin, CD31, α-SMA, and FSP-1 proteins in the myocardial tissues were quantified using Western blot analysis. As shown in Figure 7D, consistent with the results of immunofluorescence staining, after AA-NPs treatment, the fibrosis-related and EndMT-related proteins were expressed at lower levels compared to the controls. Therefore, AA-NPs interfered with the EndMT process by downregulating the expression levels of mesenchymal markers (α-SMA and FSP-1) (Figure 7E). Together, the in-depth mechanisms of AA-NPs treatment for myocardial fibrosis were associated with reducing collagen deposition, inhibiting EndMT, and improving endothelial dysfunction, and the effect was better than that of conventional treatment.

### 2.8 AA-NPs Inhibited the EndMT Process in HUVECs and HMEC-1 In Vitro

**Figure 8:**
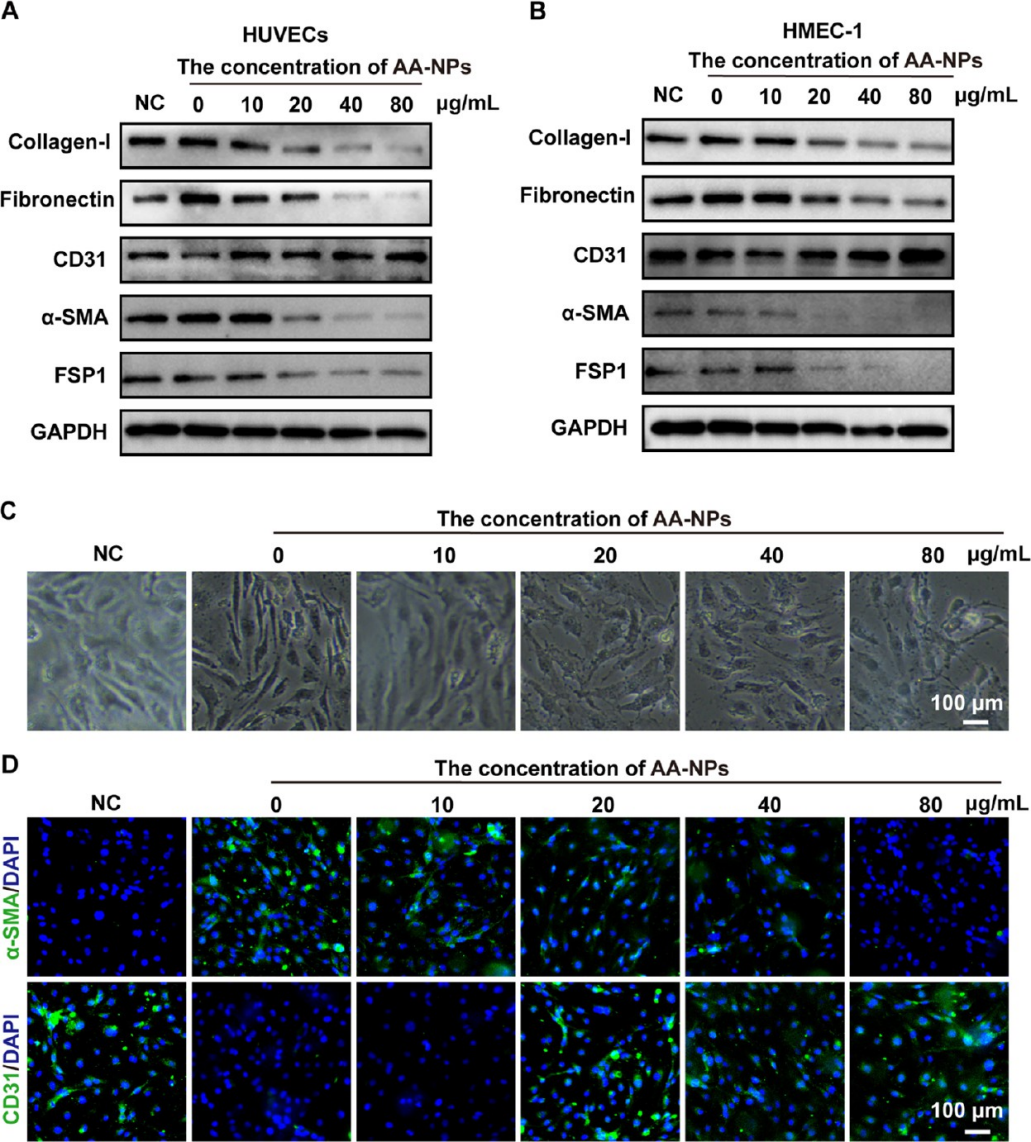
AA-NPs inhibited the EndMT process in HUVECs and HMEC-1 in vitro. After Ang II stimulation, the expression levels of collagen-I, fibronectin, CD31, α-SMA, and FSP-1 proteins in (A) HUVECs and (B) HMEC-1 treated with different concentrations of AA-NPs. (C) Morphological changes of HUVECs observed under a microscope before and after AA-NPs treatment. (D) Immunofluorescence staining of the mesenchymal marker α-SMA and vascular endothelial marker CD31 in HUVECs. All data are presented as the mean ± SD (*n* = 3).

To further investigate the inhibitory effect of AA-NPs on the EndMT process in vitro, Ang II-induced human umbilical vein endothelial cell (HUVEC) and human microvascular endothelial cells (HMEC-1) models were established. As shown in Figure 8A, after being induced by 10 μM Ang II for 48 h, we first confirmed that the expressions of collagen-I and fibronectin in HUVECs after AA-NPs treatment showed lower levels than that without treatment, indicating that AA-NPs could reduce the excessive collagen deposition. Then, it was observed that AA-NPs inhibited the expression levels of mesenchymal markers (α-SMA and FSP-1) and recovered the expression levels of the vascular endothelial marker (CD31) in HUVECs, suggesting the reduced EndMT process and restored vascular indicators. Notably, as the concentration of AA-NPs increased to 80 μg/mL, the inhibitory activity on EndMT in HUVECs became more obvious, showing a dose-dependent activity. Similarly, AA-NPs with concentrations between 10 and 80 μg/mL also had a protective effect on HMEC-1 stimulated by Ang II, as evidenced by decreased collagen markers (collagen-I and fibronectin), decreased mesenchymal markers (α-SMA and FSP-1), and increased vascular endothelial marker (CD31) (Figure 8B). EndMT is a process by which endothelial cells transform into mesenchymal cells and can be clearly observed under a microscope. In Figure 8C, normal HUVECs exhibited a cobblestone-like morphology. After being induced by Ang II, the HUVECs surface became rough and exhibited a slender spindle shape, indicating that HUVECs undergo an EndMT process. Interestingly, the spindle-like HUVECs gradually recovered to a cobblestone shape after AA-NPs treatment, suggesting that AA-NPs could inhibit EndMT in HUVECs. Subsequently, immunofluorescence staining proved that the mesenchymal marker α-SMA was upregulated, while the vascular endothelial marker CD31 was downregulated in HUVECs after Ang II stimulation (Figure 8D). After AA-NPs treatment, the downregulated α-SMA expression and upregulated CD31 expression were observed, which was consistent with the Western blot results. These results demonstrated that AA-NPs could inhibit the EndMT process at the cellular level in vitro.

## 3 Conclusions

For the first time, irregular spherical supermolecules were found in the AM–AS codecoction. AA-NPs with controlled size and rich surface functional groups showed excellent water solubility and storage stability. A total of 43 chemical components were identified in AA-NPs by UHPLC-HR-MS analysis, showing a dynamic self-assembly process. After encapsulation and release analysis, it was confirmed that eight key components of AA-NPs were effectively loaded into the nanoparticles and showed sustained release. More interestingly, LIG and AIV exhibited restricted release with a 24 h release rate below 50%, suggesting the possible interactions. Therefore, taking LIG and AIV as examples to investigate the self-assembly of AA-NPs, we confirmed that the self-assembled LIG–AIV complex was formed with a molar ratio of 2:1. Subsequently, AA-NPs were shown to be effective in mice myocardial fibrosis models induced by isoproterenol. In particular, the underlying mechanism of antifibrotic activity was related to the recovery of cardiac function, reduction of collagen deposition, and inhibition of EndMT. Meanwhile, the inhibitory effect of AA-NPs on the EndMT process *in vitro* was verified in HUVECs and HMEC-1. Overall, this study systematically characterized the basic properties, chemical compositions, and potential self-assembly mechanism of supramolecules separated from the AM–AS codecoction, providing an outstanding example to deeply understand the formation mechanism of supramolecules in herb pair decoctions and design carrier-free natural small-molecule self-assembled nanoparticles. At the same time, AA-NPs may be the chemical material basis of the molecular mechanism of AM–AS decoction in the treatment of isoproterenol-induced myocardial fibrosis.

## 4 Experimental Section

### 4.1 Materials and Reagents

*A. membranaceus* (Fisch.) *Bge.* (AM, batch number: 221212–3) and *A. sinensis* (Oliv.) *Diels* (AS, batch number: 220510–3) were purchased from Baicaotang Chinese Herbal Medicine (Luzhou, China). Astragaloside IV (AIV, CAS number: 84687–43–4) was obtained from Dalian Meilun Biotechnology Co. Ltd. (Dalian, China). Z-ligustilide (LIG, CAS number: 81944–09–4) was obtained from Sichuan Weiqi Biotechnology Co. Ltd. (Chengdu, China). Dialysis bags (MWCO: 3500 Da), ultrafiltration tubes (MWCO: 1000 Da, 3500 Da), and phosphate buffer (pH = 7.4) were provided by Solarbio Science & Technology Co. Ltd. (Beijing, China). Methanol and formic acid of mass spectrum grade were obtained from ThermoFisher Technology Co. Ltd. Isoproterenol and fosinopril sodium were purchased from MedChemExpress Co. Ltd. (New Jersey, China). Deionized water was purchased from Watsons Co. Ltd. (China).

### 4.2 Preparation of AA-NPs from the AM–AS Codecoction

According to the weight ratio of AM and AS in the *Danggui Buxue* decoction prescription, 16.7 g of AM and 3.3 g of AS were accurately weighed and soaked in 400 mL of deionized water for 30 min. After heating and boiling for 10, 20, 30, 40, 50, 60, 90, and 120 min, respectively, the obtained solution was centrifuged at 4000 rpm for 10 min to remove residues. Subsequently, the supernatant was placed in ultrafiltration tubes (MWCO: 3500 Da) and centrifuged at 12,000 rpm for 40 min. Samples trapped in the upper layer were collected and named AA-NPs. The Tyndall effect of AA-NPs was observed by using laser pointer irradiation.

### 4.3 Characterization of AA-NPs

The dynamic light scattering (DLS) method was applied to detect the size distribution of AA-NPs. The particle size, polydispersity index (PDI), and ζ-potential of AA-NPs were determined using a nanoparticle size analyzer (90plus PALS, Bruker, Germany). The morphology of AA-NPs was visualized through transmission electron microscopy (TEM, JEM2100F, JEOL, Japan) and scanning electron microscopy (SEM, SPA 300HV, SEIKO, Japan). The surface functional groups and crystallinity of AA-NPs were measured via an infrared spectrometer (IRTracer 100, Shimadzu, Japan) and X-ray diffractometer (Ultima IV, Rigaku, Japan), respectively.

### 4.4 Spectral Properties of AA-NPs

The absorption spectra and fluorescence properties of AA-NPs were observed by using a double-beam UV–vis spectrophotometer (TU-1900, Shanghai, China) and fluorescence spectrophotometer (FS5, Edinburgh, U.K.), respectively. The scanning wavelength range of the UV–vis spectrum was set as 200–800 nm. The slit for the fluorescence detection of all samples was 2 mm.

### 4.5 Storage Stability of AA-NPs

Lyophilized AA-NPs were stored at −80 °C and redissolved in deionized water on days 1, 7, 14, 21, and 30. The fluorescence intensity, particle size, PDI, and ζ-potential of AA-NPs were measured to evaluate the storage stability.

### 4.6 Chemical Component Analysis

UHPLC-HR-MS analysis was performed on a ThermoFisher UltiMate3000 UHPLC System connected with a ThermoFisher Q-Exactive Orbitrap mass spectrometer. An ACQUITY UPLC HSS T3 column (2.1 mm × 100 mm, 1.8 μm, Waters, Ireland) was used for chemical component separation. The mobile phase consisted of components A (water containing 0.1% formic acid) and B (methanol). The gradient elution conditions were as follows: 0–5 min, 5% B; 5–10 min, 5–20% B; 10–30 min, 20–55% B; 30–50 min, 55–80% B; 50–56 min, 80–100% B; 56–58 min, 100% B; 58–58.5 min, 100–5% B; 58.5–60 min, 5% B. The flow rate and injection volume were 0.2 mL/min and 5 μL, respectively. Meanwhile, an electron spray ion (ESI) source was applied to obtain the mass spectra of positive and negative ion modes in the *m*/*z* range of 100–1500 Da. The ESI parameters were as follows: spray voltage: 3.5 kV; capillary temperature: 350 °C; and sheath gas flow rate: 5 L/min. After centrifugation at 12,000 rpm for 20 min, AA-NP solutions were directly injected for UHPLC-HR-MS analysis. Compound identification was performed using Xcalibur 4.5 software (ThermoFisher Scientific), and the mass tolerance was set as 5 ppm.

### 4.7 Encapsulation Efficiency of AA-NPs

The encapsulation efficiencies of AA-NPs of eight main components, including astragaloside IV, formononetin, ononin, calycosin, calycosin-7-glucoside, ferulic acid, Z-ligustilide, and senkyunolide I, were determined by the ultrafiltration–centrifugation method. In brief, 0.5 mL of the AA-NPs sample was placed into an ultrafiltration tube (MWCO: 3500 Da) and centrifuged at 12,000 rpm for 30 min. Then, the upper and lower samples of the tube were collected separately. Samples before centrifugation and the upper layer after centrifugation were analyzed by UHPLC-HR-MS. The encapsulation efficiency of AA-NPs was calculated. Three batches of samples were tested in parallel.

### 4.8 In Vitro Release of AA-NPs

The dialysis method was used to investigate the eight main components released from AA-NPs. Eight milliliters of the AA-NPs sample was put into a dialysis bag (MWCO: 3500 Da), immersed in 160 mL release medium of phosphate buffer (pH = 7.4), and shaken at a speed of 100 rpm at 37 °C for 12 h. Meanwhile, 1 mL of the release medium was taken out at 0.083, 0.167, 0.5, 1, 2, 4, 6, 8, 10, and 12 h, respectively. Then, 1 mL of fresh PBS was added. Five microliters of the sample were injected and analyzed by UHPLC-HR-MS. Three batches of samples were tested in parallel. Finally, the cumulative release percentage of each component was calculated. All results were repeated in three parallel measurements.

### 4.9 Preparation and Characterization of the Self-Assembled LIG–AIV Complex

The LIG–AIV self-assembled complex was prepared using a one-step ultrasonic method.^26^ In brief, certain molar ratios of LIG and AIV (1:1, 2:1, 4:1, 1:0, and 0:1) were designed following previous reports with some modifications.^31^ Subsequently, the mixtures were sonicated by an ultrasonic probe under 200 W power for 15 min to prepare the self-assembly system. The LIG–AIV self-assembled complexes were named LV 1–1, LV 2–1, and LV 4–1, corresponding to LIG–AIV molar ratios of 1:1, 2:1, 4:1, 1:0, and 0:1, respectively. The morphologies of LV 1–1, LV 2–1, LV 4–1, and LIG were visualized through SEM. In addition, a double-beam UV–vis spectrophotometer, fluorescence spectrophotometer, and infrared spectrometer were applied to indicate the interaction between LIG and AIV. UHPLC-HR-MS analysis was performed to determine the exact mass number and binding ratio of the LIG–AIV self-assembly complex.

### 4.10 Molecular Dynamics Simulations

As previously reported, molecular dynamics simulations of interactions between AIV and LIG were performed using Amber 20 and AmberTools 20.^32^ The initial 2D structures of LIG and AIV were obtained from the PubChem database (https://pubchem.ncbi.nlm.nih.gov/). The molecular dynamics process consisted of an equilibrium phase and a production phase, which were randomly assigned to different initial speeds according to the Maxwell–Boltzmann distribution.^21^ During the simulation, a 44.102 Å × 45.373 Å × 35.186 Å box was constructed and randomly filled with two LIG molecules and one AIV molecule to form the simulation system. The energy interval of the entire system was run for 1000 steps. The simulation was performed for 100 ns with a time step of 0.002 ps and a nonbonding cutoff of 10 Å. The trajectory interval was set as every 5000 steps for 10 ps. In the production-phase simulation, snapshots were captured every 20 ns.

### 4.11 In Vitro Release of the Self-Assembled LIG–AIV Complex

The dialysis method was used to investigate the release kinetics of LIG and AIV from the self-assembled LIG–AIV complex in different pH environments. In brief, 4 mL of the LV 2–1 sample was put into a dialysis bag (MWCO: 3500 Da), immersed in 100 mL of release medium of phosphate buffer (pH = 5.2, 6.8, and 7.4), and shaken at a speed of 100 rpm at 37 °C for 48 h. Meanwhile, 1 mL of the release medium was taken out at 0.083, 0.167, 0.5, 1, 2, 4, 6, 8, 10, 12, 24, and 48 h, respectively. Then, 1 mL of fresh PBS was supplemented. Five microliters of the sample were injected and analyzed by UHPLC-HR-MS. Three batches of samples were tested in parallel. Finally, the cumulative release percentage of each component was calculated. All results were repeated in three parallel measurements.

### 4.12 Therapeutic Efficacy of AA-NPs in Myocardial Fibrosis

C57BL/6J male mice (8–10 weeks, 22 ± 2 g) were purchased from GemPharmatech (Chengdu, China). All animal welfare and experimental procedures were strictly in accordance with the guide for the care and use of laboratory animals and approved by the animal care and use committee of Southwest Medical University, Luzhou, China (Approval No. 20221116–005). All animals were kept at a room temperature of 25 ± 2 °C and a relative humidity of 60 ± 5%. After 3 days of adaptive feeding, mice were randomly divided into five groups: normal group (NC, *n* = 4), isoproterenol group (ISO, *n* = 4), fosinopril sodium group (FOS, *n* = 4), low-dose AA-NP group (L-AA, *n* = 4), and high-dose AA-NP group (H-AA, *n* = 4). An isoproterenol-induced myocardial fibrosis (MF) model was established, as previously reported.^33^ In brief, C57BL/6j mice were injected intraperitoneally with isoproterenol (5 mg/kg) once a day for 28 days. Meanwhile, the L-AA and H-AA groups were injected intraperitoneally each day at doses of 20 and 80 mg/kg, respectively. The NC group was given the same amount of normal saline. FOS was selected as the positive control drug and injected intraperitoneally at a dose of 2.4 mg/kg/d. On day 28, mice were anesthetized with 3% isoflurane. Cardiac parameters were determined on an ultrahigh-resolution small-animal ultrasound imaging machine (Vevo 3100, FUJIFILM VisualSonics, Canada). The ejection fraction (EF%), fractional shortening (FS%), and left ventricular end-systolic diameter (LVESD) were calculated.^27^ The cardiac tissues were fixed with paraformaldehyde and stained with hematoxylin–eosin (H&E), Sirius red, and Masson to observe the pathological features and collagen fiber distribution, respectively. In addition, immunofluorescence staining of paraffin-embedded cardiac tissues was performed using antibodies against collagen-I, α-SMA, fibronectin, and FSP-1. A Western blot analysis was performed to quantify collagen-I, fibronectin, CD31, α-SMA, and FSP-1 protein expression levels in the myocardial tissue samples according to standard procedures.

### 4.13 In Vitro Cell Experiments

Human umbilical vein endothelial cells (HUVEC) and human microvascular endothelial cell line-1 (HMEC-1) were purchased from iCell Bioscience Inc. (Shanghai, China) and cultured in DMEM (Gibco) containing 10% fetal bovine serum (Gibco) and 1% penicillin–streptomycin (Gibco). As previously reported, the in vitro EndMT model was established by 10 μM Ang II stimulation.^34^ In detail, HUVECs (or HMEC-1) were cultured to a density of 50%, starved in a medium containing 3% FBS for 12 h, and then stimulated with Ang II for 48 h. The expression levels of collagen-I, fibronectin, CD31, α-SMA, and FSP-1 proteins in HUVECs and HMEC-1 treated with different concentrations of AA-NPs were detected by Western blotting. Besides, the morphology of HUVECs was observed under a microscope (ECLIPSE Ts2R, Nikon, Japan). Immunofluorescence staining of HUVECs was performed using antibodies against α-SMA and CD31 according to standard protocols.

### 4.14 Statistical Analysis

All data are presented as the mean ± SD. The statistical significance between two groups was analyzed using Student’s test or one-way ANOVA multiple comparisons. Figures and graphs were constructed by using Adobe Illustrator 2023 and OriginPro 9.1 software.

## Supporting Information Available

Fluorescence intensities of LIG, LV 1–1, LV 2–1, and 4–1 (Figure S1); molecular simulation of the three-dimensional configuration of LIG and AIV (Figure S2); identification of 43 components in AA-NPs by UHPLC-HR-MS in positive and negative ion modes (Table S1) (PDF)
