# Supplementary material for: Insights into the Mechanism of Supramolecular Self-Assembly in the Astragalus membranaceus–Angelica sinensis Codecoction
Source: ACS Appl Mater Interfaces. 2023 Oct 4;15(41):47939–54. doi: 10.1021/acsami.3c09494 (PMC10591233; doi:10.1021/acsami.3c09494)
Supplement: Supplementary file 1 — am3c09494_si_001.pdf [file am3c09494_si_001.pdf]

Supporting Information

The insight mechanism of supramolecular self-assembly in the *Astragalus membranaceus*-*Angelica sinensis* codecoction

Pan Liang[1,2,+], Tao Bi[2,+], Yanan Zhou[2], Yining Ma[2], Xinyue Liu[2], Wei Ren[2], Sijin Yang[2,], Pei Luo[1,*]*

[+] These authors contributed equally to this work and were co-first authors.

[1] State Key Laboratories for Quality Research in Chinese Medicines, Macau University of Science and Technology, Macau 999078, China.

[2] National Traditional Chinese Medicine Clinical Research Base and Drug Research Center of Integrated Traditional Chinese and Western Medicine, the Affiliated Traditional Chinese Medicine Hospital of Southwest Medical University, Luzhou 646000, China.

*** Correspondence:**

Pei Luo,

State Key Laboratories for Quality Research in Chinese Medicines, Macau University of Science and Technology, Macau 999078, China.

E-mail address: pluo@must.edu.mo

Sijin Yang,

National Traditional Chinese Medicine Clinical Research Base and Drug Research Center of Integrated Traditional Chinese and Western Medicine, the Affiliated Traditional Chinese Medicine Hospital of Southwest Medical University, Luzhou 646000, China.

E-mail address: ysjimn@sina.com

Coauthor e-mail address: xnydzyylp@swmu.edu.cn (P. Liang), bitao456321@163.com (T. Bi), 18703667421@163.com (Y. Zhou), 20210399120027@stu.swmu.edu.cn (Y. Ma), lxinyue1119@163.com (X. Liu), renwei1991@swmu.edu.cn (W. Ren), ysjimn@sina.com (S. Yang), pluo@must.edu.mo (P. Luo).

FIGURE SECTION

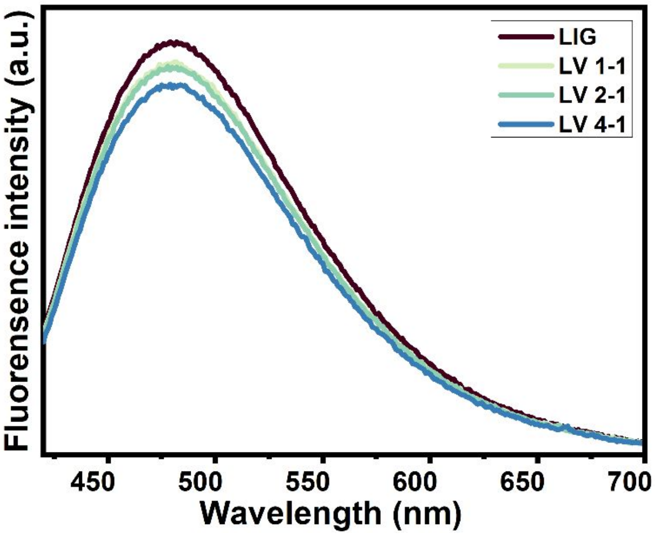

Figure S1 Fluorescence intensity of LIG, LV 1-1, LV 2-1 and 4-1.

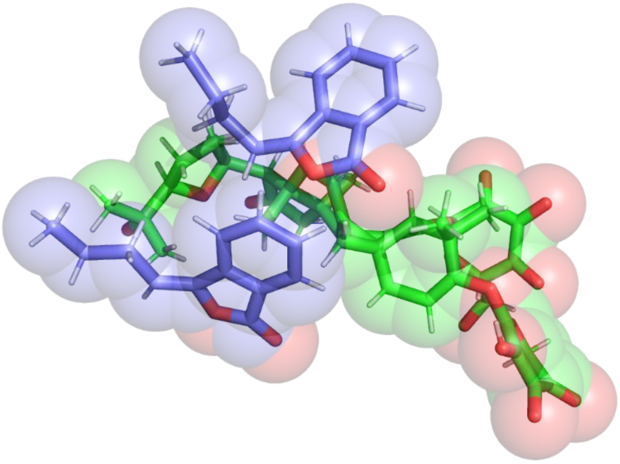

Figure S2. Molecular simulation of the three-dimensional configuration of LIG and AIV.

TABLE SECTION

Table S1. Identification of 43 components in AA-NPs by UHPLC-HR-MS in positive and negative ion modes.

No	tR (min)	Identification	Formula	Measured (m/z)	Ion form	Error (ppm)	Fragment ion (m/z)	Peak area	Peak area ratio (%)	Classification
1	1.46	Arginine	$C_6H_{15}N_4O_2$	175.11815	$[M+H]^+$	-0.802	158.09186, 141.06511, 130.09712, 116.07039, 112.08677	33009574575	14.49724613	Amino acids
2	2.13	Sucrose	$C_{12}H_{22}O_{11}Na$	365.10327	$[M+Na]^+$	1.855	203.05203, 185.04156, 173.02879, 161.02928, 143.01866, 119.01862, 101.00867	3844477540	1.688429428	Others
3	2.64	Citric acid	$C_6H_7O_7$	191.01813	$[M-H]^-$	0.11	173.00761, 154.99689, 147.02846, 129.01770, 111.00712, 101.02274	22210380325	9.754422899	Organic acids
4	3.21	Glutathione disulfide	$C_{13}H_{33}O_{20}N_5S$	611.14337	$[M-H]^-$	0.032	524.94916, 338.04758, 306.07559, 288.06604, 272.08817, 254.07730, 210.08728, 179.04457, 160.00574, 143.04454, 128.03362	622808403	0.273526903	Amino acids
5	4.62	Adenosine	$C_{10}H_{14}O_4N_5$	268.10223	$[M+H]^+$	2.423	136.06099	2715250532	1.19249205	Others
6	5.35	Guanosine	$C_{10}H_{14}O_5N_5$	284.09702	$[M+H]^+$	2.129	152.05571, 135.02930	1154942868	0.507231348	Others

7	5.38	Guanine	$C_5H_6ON_5$	152.05569	$[M+H]^+$	2.326	135.02934, 128.04475, 110.03448, 104.39621	5666068972	2.488441536	Others
8	11.62	Indole-3-acrylic acid	$C_{11}H_{10}O_2N$	188.06909	$[M+H]^+$	1.993	170.05891, 160.07452, 146.05904, 118.06454, 115.05366	6978523817	3.064849477	Organic acids
9	13.48	5-Methoxy-L-tryptophan	$C_{12}H_{13}O_2N_2$	217.09581	$[M-H_2O+H]^+$	2.652	171.09067, 144.07997, 132.08014, 127.05362, 117.06944	855690377	0.375804722	Amino acids
10	14.70	Umbelliferone	$C_9H_7O_3$	163.03821	$[M+H]^+$	1.959	145.02791, 135.04356, 117.03324, 107.04906	553273700	0.242988439	Others
11	20.61	Ferulic acid	$C_{10}H_9O_4$	193.04886	$[M-H]^-$	-3.498	178.02541, 149.05896, 134.03554, 121.02765, 106.04063	606592867	0.266405314	Organic acids
12	21.31	Calycosin-7-O-β-D-glucoside	$C_{22}H_{23}O_{10}$	447.12537	$[M+H]^+$	-6.493	285.07401, 270.05075, 253.04810, 225.05338, 214.06120, 197.05862, 137.02263	9929236153	4.360752364	Flavonoids
13	24.04	3-Hydroxy-9-methoxyptercarpan	$C_{16}H_{15}O_5$	287.1698	$[M+H]^+$	1.498	153.05475, 105.97922	221040	9.7077E-05	Flavonoids

14	24.63	4-hydroxy-3-n-butylphthalide	$C_{12}H_{15}O_3$	207.09991	$[M+H]^+$	0.189	189.08981, 179.10538, 165.08995, 161.09508, 147.07942, 133.06403, 119.08498, 105.06953	5928185620	2.603558729	Others
15	24.67	Senkyunolide I	$C_{12}H_{16}O_4Na$	247.09204	$[M+Na]^+$	-0.163	195.24634, 175.15773, 168.67059, 125.88958, 111.10343	2650721553	1.164152015	Phthalides
16	26.50	Ononin	$C_{22}H_{23}O_9$	431.13068	$[M+H]^+$	0.583	355.59268, 269.07916, 254.05585, 237.05327, 213.08974, 197.05844, 154.02509, 137.02264, 118.04084	5768926385	2.53361477	Flavonoids
17	26.62	Azelaic acid	$C_9H_{15}O_4$	187.09599	$[M-H]^-$	-0.083	169.08563, 143.10594, 137.50829, 125.09549, 111.65796	3235433283	1.420947505	Organic acids
18	26.87	Rhamnocitrin	$C_{17}H_{17}O_5$	301.10483	$[M+H]^+$	0.531	-	4240463291	1.862339664	Flavonoids
19	27.63	Calycosin-7-O-Glc-6"-O-acetate	$C_{24}H_{25}O_{11}$	489.13638	$[M+H]^+$	2.969	285.07404, 270.05078, 253.04810, 225.05338, 214.06129, 183.04306, 137.02263	1075755676	0.472453675	Flavonoids
20	27.66	6″-O-malonate-calycosin-7-O-β-D-glucoside	$C_{25}H_{25}O_{13}$	533.12891	$[M+COOH]^-$	-0.801	283.06039, 268.03702, 239.03409, 211.03856, 184.05116, 148.01447	378268164	0.166128972	Flavonoids

21	28.91	Isomucronulatol-7-O-β-D-glucoside	$C_{23}H_{27}O_{10}$	463.15997	$[M-H]^-$	2.324	301.10751, 286.08398, 271.06064, 256.03711, 227.06966, 179.07005, 164.04643, 149.02289, 135.04361, 121.02793	1227764668	0.53921345	Flavonoids
22	29.74	Dihydroxy-methoxyisoflavone	$C_{16}H_{13}O_5$	285.07327	$[M+H]^+$	0.211	270.05060, 253.04797, 225.05327, 213.05334, 197.05853, 186.06654, 137.02254, 134.03552	18939754595	8.318019468	Flavonoids
23	29.75	Calycosin	$C_{16}H_{13}O_5$	285.07358	$[M+H]^+$	-7.928	270.05060, 253.04797, 225.05327, 213.05334, 197.05853, 186.06654, 137.02254, 134.03552	18939869308	8.318069848	Flavonoids
24	30.39	Hydroxy-methoxyisoflavone	$C_{16}H_{11}O_4$	267.0657	$[M-H]^-$	2.976	252.04234, 223.03931, 208.05196, 195.04408, 180.05681, 153.01836, 135.00725, 132.02029	1346985092	0.591573041	Others
25	30.39	Formononetin-7-O-Glc-6''-O-malonate	$C_{25}H_{25}O_{12}$	517.13025	$[M+H]^+$	1.136	269.07898, 254.05571, 237.05304, 213.08963, 197.05818, 154.02515, 118.04076, 107.04879	1795897529	0.788727781	Flavonoids

26	32.42	Formononetin-7-O-Glc-6"-O-acetate	$C_{24}H_{25}O_{10}$	473.14056	$[M+H]^+$	1.874	455.34875, 323.27338, 269.07910, 254.05569, 237.05312, 213.08969, 197.05814, 154.02539	696136756	0.305731474	Flavonoids
27	32.87	3-Hydroxy-9,10-dimethoxyptercarpan	$C_{17}H_{17}O_5$	301.10474	$[M+H]^+$	0.531	269.07904, 241.08435, 191.06900, 167.06921, 152.04588, 147.04315, 134.03546, 123.04346, 106.04098	6312290155	2.772250936	Others
28	34.44	Isomucronulatol	$C_{17}H_{19}O_5$	303.1207	$[M+H]^+$	1.055	193.08513, 181.08501, 167.06949, 161.05898, 152.04617, 133.06421, 123.04363, 105.06973	1555846496	0.683301432	Flavonoids
29	35.25	Formononetin	$C_{16}H_{13}O_4$	269.07877	$[M+H]^+$	0.24	254.05638, 237.05365, 226.06171, 213.09026, 197.05904, 181.06432, 154.02553, 137.02283, 118.04111	15784103296	6.932110859	Flavonoids
30	35.60	Senkyunolide A	$C_{12}H_{17}O_2$	193.12119	$[M+H]^+$	1.469	175.11087, 147.11610, 137.05907, 123.04358, 119.08517, 109.06458, 105.06969	1731577240	0.760479399	Phthalides

31	38.27	E-ligustilide	$C_{12}H_{15}O_2$	191.10509	$[M+H]^+$	0.072	173.09496, 163.11060, 155.08446, 149.05875, 145.10022, 130.07695, 117.06932, 105.06950	4765198315	2.0927944	Phthalides
32	39.52	Z-ligustilide	$C_{12}H_{15}O_2$	191.1051	$[M+H]^+$	1.334	173.09509, 163.11079, 155.08463, 149.05887, 145.10033, 130.07703, 117.06941, 105.06959	32238203666	14.15847309	Phthalides
33	46.68	Soyasaponin I	$C_{48}H_{79}O_{18}$	943.51874	$[M+H]^+$	2.298	599.38977, 441.37009, 423.35947, 405.34924, 305.08414, 247.20416, 217.19373, 203.17827, 141.01736	264570872	0.116195046	Saponins
34	47.25	Astragaloside V	$C_{48}H_{79}O_{21}$	991.51147	$[M+COOH]^-$	0.64	945.50537, 783.45819, 613.12482, 551.26764, 391.65607	293985345	0.129113385	Saponins
35	48.39	Astragaloside Ⅳ	$C_{42}H_{69}O_{16}$	829.4585	$[M+COOH]^-$	0.588	783.45978	1440495410	0.632641189	Saponins
36	48.85	Astragaloside Ⅱ	$C_{43}H_{70}O_{15}Na$	849.45569	$[M+Na]^+$	0.503	764.06604, 669.39478, 609.37408, 477.33069, 255.24699, 203.05202	1178721883	0.517674689	Saponins
37	49.08	Astramembrannin II	$C_{35}H_{58}O_9Na$	645.39374	$[M+Na]^+$	1.372	546.09784, 437.56229, 101.87804	202477816	0.088924828	Saponins
38	49.44	Astragaloside ⅡI	$C_{41}H_{68}O_{14}Na$	807.44806	$[M+Na]^+$	-2.487	627.38464	12862554	0.005649016	Saponins

40	50.57	Cyclocephaloside Ⅱ	$C_{44}H_{71}O_{17}$	871.46759	$[M+COOH]^-$	-0.926	825.45868, 765.43420, 456.71658, 189.68484, 161.04445, 128.68369, 113.02245, 101.02254	828830179	0.364008178	Saponins
39	50.57	Isoastragaloside Ⅱ	$C_{44}H_{71}O_{17}$	871.46759	$[M+COOH]^-$	-2.188	825.45935, 460.65497, 414.71649, 231.30002, 179.05495, 119.03292	1131263289	0.496831678	Saponins
41	51.68	Astragaloside Ⅰ	$C_{45}H_{72}O_{16}Na$	891.46356	$[M+Na]^+$	0.609	831.44794, 711.40369, 651.38214, 591.36432, 477.33289, 323.98587	1318818628	0.579202807	Saponins
42	52.47	Neoastragaloside I	$C_{46}H_{73}O_{18}$	913.47736	$[M+COOH]^-$	-5.224	788.98376, 750.94653, 385.21240, 269.30359, 226.73837, 169.92935, 149.47536	2925107391	1.284657629	Saponins
43	54.19	Isoastragaloside Ⅰ	$C_{46}H_{73}O_{18}$	913.47809	$[M+COOH]^-$	-1.151	867.46637, 832.82928, 609.54138, 428.03296, 400.55896, 250.06491, 177.27364	1339927278	0.588473368	Saponins
